# Chicago Medical Society

**Published:** 1888-06

**Authors:** 


ANNUAL MEETING, APRIL 2, 1888.

The President, Wm. T. Belfield, in the chair.

The minutes of the last regular meeting were read and approved.

The annual reports of the secretary and treasurer, and of the auditing committee and committees on library and publication were read and severally accepted by the society.

President Belfield then delivered a brief address and offered some practical suggestions regarding the future work of the society.

He advised that the society adopt, in the near future, the plan of organization of the New York Academy of Medicine, namely, the organization of sections, one for each important subdivision—internal medicine, surgery, gnyæcology, etc. Each section to have its own officers and organization and hold independent meetings, which all members of the entire society have the privilege of attending; while the general society meeting should occur once each month. By this plan all the benefits of special societies are secured to specialists, with the additional advantage that their work is accessible to those who are not specialists—an exchange of mutual value. As the members of our local special societies are, almost without exception, members of the Chicago Medical Society also, this extension of the work could be accomplished without serious difficulty.

Two other subjects, of vital interest to the profession at large, should receive the immediate and earnest attention of the society:

First, it should be impressed upon the trustees of the Newberry Library Fund that that library—intended for general scientific as well as literary reference—should be equipped with files of the standard medical periodicals of all nations. We have long been aware of the dearth of medical literature in this city, and have made several attempts, through our library committee, to supply this demand. The small degree of success attained has been due chiefly to lack of money. Of this the Newberry Fund has plenty, and a very small fraction of the amount to be expended for literature will secure what is needed. Since this is one of the legitimate objects of the library, it is, probably, only necessary that the matter be properly brought before the trustees to secure their cordial assent.

The second object which this society can properly and profitably strive to attain, is the withdrawal of the licensing power in this State from the medical colleges, and its investment in a board of examiners free from all college affiliations. Of the desirability of the change there is but one opinion among disinterested persons, especially since the advantages of the plan have been amply demonstrated under European governments as well as in several of our States. It seems eminently appropriate that this society—the largest in the State—should inaugurate a movement which must redound to the advantage of all reputable physicians in Illinois. The only opposition to be expected is by interested parties, particularly members of medical college faculties; and it is only just to state that these gentlemen are by no means a unit—in this city at least—in opposing the plan.

He regarded it as a pleasure as well as a duty to acknowledge that whatever of success attended the direction of the society's affairs during the past year had been largely due to the judgment and zeal of its secretary.

The election of officers for the ensuing year resulted as follows: J. H. Etheridge, president; A. E. Hoadley, first vice-president; R. D. MacArthur, second vice-president; Frank Billings, secretary; Frank S. Johnson, treasurer; J. H. Wilson, necrologist; Wm. E. Quine, member of committee on judiciary; Liston H. Montgomery, member of committee on membership; John Bartlett, member of committee on library; Wm. T. Thackeray, member of the committee on publication.

On motion an amendment to Art. IV of the By-laws was made so as to read: "The annual dues shall be three dollars."

A motion was made and seconded to adopt the following resolution: "That the committee on publication be instructed to publish monthly in pamphlet form the transactions of the society, and send a copy, by mail, to each member of the society and the prominent medical journals; the expense for such publication to be defrayed according to the provisions of Art. IV, Sec. 11 of the Constitution."

A motion was made, and carried, to amend the resolution, limiting the expense of such publication of the transactions to a sum not to exceed twenty dollars per month.

The amended resolution was then unanimously adopted.

